# Interactions between intestinal pathogens, enteropathy and malnutrition in developing countries

**DOI:** 10.1097/QCO.0000000000000261

**Published:** 2016-04-27

**Authors:** Andrew J. Prendergast, Paul Kelly

**Affiliations:** a Blizard Institute, Queen Mary University of London, London, UK; b Zvitambo Institute for Maternal and Child Health Research, Harare, Zimbabwe; c Department of International Health, Johns Hopkins Bloomberg School of Public Health, Baltimore, Maryland, USA; d University Teaching Hospital, Lusaka, Zambia

**Keywords:** child, diarrhoea, enteropathy, malnutrition, microbiota

## Abstract

**Purpose of review:**

This review focuses on recent data highlighting the interactions between intestinal pathogens, enteropathy and malnutrition in developing countries, which drive morbidity and mortality and hinder the long-term developmental potential of children.

**Recent findings:**

Diarrhoea remains the second commonest cause of death in children below 5 years, and malnutrition underlies 45% of all child deaths. Even in the absence of diarrhoea, subclinical pathogen carriage and enteropathy are almost universal in developing countries. Here, we review recent studies addressing the causes and consequences of diarrhoea; emerging data on environmental influences that govern postnatal development of the gut and microbiota; current concepts of environmental enteric dysfunction; and recent intervention trials in the field. We highlight the interactions between these processes, whereby intestinal pathogens drive a cycle of gut damage, malabsorption, chronic inflammation and failed mucosal regeneration, leading to malnutrition and susceptibility to further enteric infections.

**Summary:**

Efforts to improve child survival and long-term developmental potential need to address the overlapping and interacting effects of diarrhoea, enteropathy and malnutrition. Recent insights from human and animal studies suggest potential targets for intervention.

## INTRODUCTION

Malnutrition underlies 45% of child deaths globally. Stunting is the commonest presentation of malnutrition, affecting approximately one-third of children in developing countries, leading to increased mortality from infections such as diarrhoea. Although there have been huge reductions in diarrhoeal mortality over several decades, diarrhoea remains the second commonest cause of death among children below 5 years. Precise mortality estimates vary between 666 000 and 712 000 annually [1], with young children (<2 years) accounting for the majority of deaths. Even in the absence of diarrhoea, subclinical pathogen carriage and enteropathy are almost universal in developing countries. This review will focus on the interactions between intestinal pathogens, enteropathy and malnutrition, drawing on recent findings from human and animal studies.

### Causes of diarrhoea

Two major studies have recently shed light on the cause of diarrhoea in developing countries. The Global Enteric Multisite Study (GEMS) [2^▪▪^] enrolled children aged 0–59 months with moderate-severe diarrhoea (MSD) at seven sites in Africa and Asia. Conventional culture, immunoassays and multiplex PCR were used to comprehensively identify diarrhoeal pathogens. A major strength was the careful design, in which one to three matched controls were recruited per case, to calculate adjusted population attributable fractions, which account for asymptomatic colonization. Overall, potential pathogens were identified in 83% of children with diarrhoea and 72% of controls, highlighting the frequency of enteropathogen carriage. Four pathogens (rotavirus, *Cryptosporidium*, *Shigella* and Enterotoxigenic *Escherichia coli* producing heat-stable toxin) were associated with MSD at all sites. Rotavirus was the commonest pathogen in infancy at all sites, *Cryptosporidium* was the second commonest cause in infants at most sites and *Shigella* became more common beyond infancy. *Giardia* was protective against diarrhoea; a smaller study from Tanzania [3] similarly reported higher *Giardia* prevalence in controls than cases [odds ratio (OR) 1.8, 95% confidence interval (CI) 1.1–3.1].

The Malnutrition and the Consequences for Child Health and Development (Mal-ED) study [4^▪▪^] had a similar diagnostic and analytic approach but focused on community rather than facility-based diarrhoea. Twice-weekly home visits to 2145 children aged 0–24 months enabled frequent collection of diarrhoeal and non-diarrhoeal specimens. Enteropathogen infection was common in children without diarrhoea, as in GEMS, from early infancy. Although one or more pathogens were detected in 76.9% of diarrhoea specimens, 64.9% of nondiarrhoea specimens had pathogens present, such that overall only 19.1% (16.2–21.8) and 33.1% (29.0–36.7) of diarrhoeal episodes in the first and second year of life, respectively, had a pathogen-specific cause determined.

There were similarities and differences to GEMS in the pathogens identified (Table 1) [2^▪▪^,^4^^▪▪^], but a relatively small number of organisms caused the burden of diarrhoea. Both GEMS and Mal-ED suggest that the global burden of cryptosporidiosis has been underestimated to date. In GEMS, *Cryptosporidium* was a significant pathogen at all sites regardless of HIV status, the second commonest pathogen in infants and was associated with subsequent mortality in 12–23-month-old children. A smaller case–control study from Tanzania [3] found a comparable prevalence to GEMS using PCR (16.3% cases), and identified HIV infection, stunting and rainy season as risk factors. An excellent recent review [5] highlights research priorities to improve prevention and treatment of cryptosporidiosis. A new tractable platform for experimental studies of *Cryptosporidium* is a major development in the field [6^▪^]. 

**Box 1:**
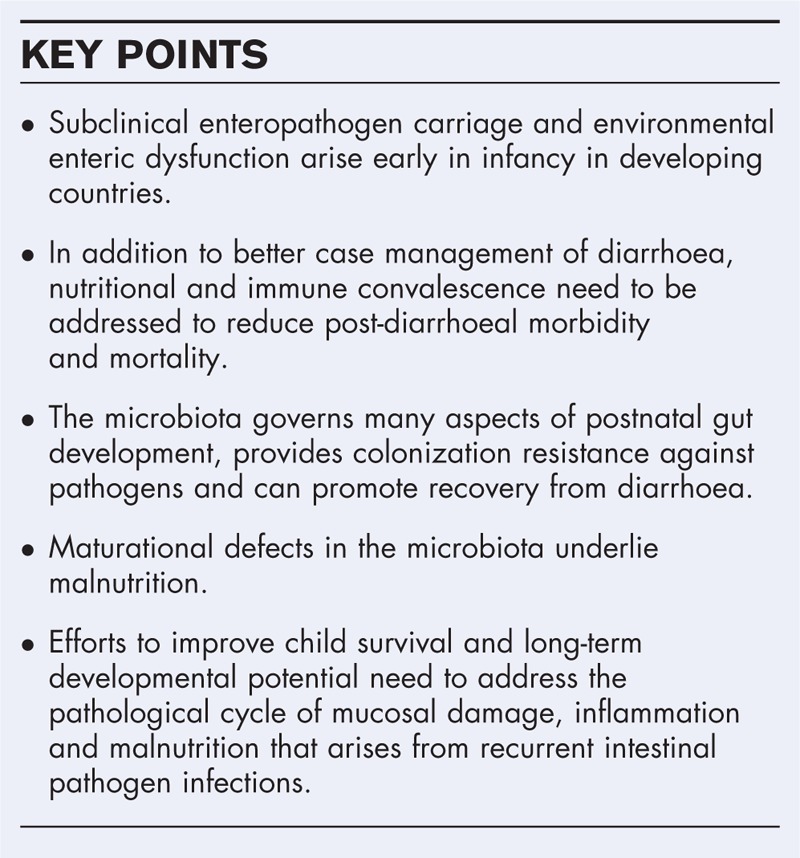
no caption available

### Consequences of diarrhoea

Despite downward trends in diarrhoeal mortality, there are still an unacceptably high number of child deaths annually. The principles of acute treatment are continued feeding, increased fluids (including oral rehydration solution), zinc supplementation and rational use of antibiotics. However, in a study from Dhaka, only 6% of caregivers of children with diarrhoea sought help from a qualified healthcare provider [7]. Even when caregivers do seek appropriate help, healthworkers may have inadequate knowledge [8] and incorrect practices are common [9]. In a survey of 264 healthcare workers in Indian slums [10], overuse of antibiotics and intravenous rehydration was widespread; practitioner knowledge strongly predicted correct practice, suggesting the need for ongoing caregiver and healthworker education.

Although most deaths arise from acute diarrhoea, a survey across seven countries found that persistent diarrhoea caused 30% or more infant diarrhoeal deaths in Ethiopia, Uganda, Tanzania, Pakistan and India [11]. Over 40% of those dying of persistent diarrhoea were severely malnourished, highlighting the interactions between diarrhoea and malnutrition; it remains unclear why some children, but not others, develop persistent diarrhoea. GEMS reported more than eight-fold increased deaths among cases compared with controls 2–3 months after a single episode of MSD (OR 8.5, 95% CI 5.8, 12.5), highlighting the neglected post-discharge mortality associated with diarrhoea, particularly among malnourished children [2^▪▪^]. Immune function may also be impacted by diarrhoea. Leptin levels during acute cholera were low and stayed suppressed for at least 1 month after recovery; leptin concentrations on day 2 were related to immunoglobulin G antibody levels to cholera toxin 30 days later, indicating the impact of leptin on immune function [12]. Together, these findings suggest that nutritional and immune convalescence need to be addressed to reduce post-diarrhoeal morbidity and mortality.

It has been debated how much diarrhoea contributes to growth failure in children. Overall, prior studies suggest a small but measurable effect on linear growth because of catch-up growth between episodes. A recent multicountry study [13] of 1007 children with longitudinal anthopometry and diarrhoeal surveillance from birth to 24 months confirmed that diarrhoea slows ponderal and linear growth, more in boys than girls. Faster (i.e. catch-up) length growth was observed during subsequent diarrhoea-free periods, confirming that catch-up growth can allow children to regain their original trajectory after short-term growth insults. Some pathogens may impair growth more than others; for example, in a Peruvian study [14], *Shigella* was particularly implicated. Although the reasons for reduced growth are multifactorial, a recent Zimbabwean study showed that diarrhoea can directly reduce circulating levels of insulin-like growth factor-1 [15].

It has been proposed that diarrhoea may adversely affect long-term neurodevelopment, although previous studies suggest this relationship arises predominantly through stunting. However, a recent study [16] of 422 Indian children, evaluated twice-weekly for illness and subsequently assessed for neurodevelopment using the Ages and Stages Questionnaire (ASQ-3), found that the number of diarrhoea days between 6 and 30 months was inversely associated with ASQ-3 scores, independent of growth. It is plausible that different pathways link diarrhoea with growth and brain development, although further mechanistic studies are needed.

### Environmental enteric dysfunction

Stunting is driven by complex interactions between genetics, epigenetics, environmental influences, recurrent infections and inadequate diet. A condition called environmental enteric dysfunction (EED), which is almost universal in impoverished settings, is also associated with stunting. EED is characterized by small intestinal inflammation and abnormal villous architecture, modest malabsorption and gut permeability; however, there is no case definition or gold standard biomarker and its cause remains unclear [17]. A recent murine model [18^▪▪^] provides insights into the interactions between microbial exposure, enteropathy and malnutrition. Mice fed a suboptimal diet developed shifts in the small intestinal microbiota but retained normal intestinal histopathology; if they also received a bacterial cocktail they developed villous blunting and inflammation characteristic of EED. This supports the hypothesis in humans that EED arises from exposure to environmental microbes in conditions of poor sanitation and hygiene, particularly in the context of inadequate diet. Frequent enteropathogen carriage indicates that environmental contamination begins early in life. Exposure to faecal bacteria through geophagia [19] and contact with animal faeces [20] may be particularly important. The hypothesized causal pathway from EED to stunting is through malabsorption and chronic inflammation (arising from microbial translocation across an impaired gut barrier); however, this is difficult to confirm with current biomarkers [17]. Recent studies using anti-endotoxin antibodies (EndoCAb) as markers of microbial translocation showed no relationships with growth in Malawi [21] or Zimbabwe [22], and plasma concentrations of intestinal fatty acid binding protein (indicative of villous damage) were elevated in Zimbabwean infants but not associated with stunting [22]; however, the role of chronic inflammation in stunting has been confirmed in several recent studies [22,23]. Dissecting the interactions between recurrent infections, impaired gut integrity, chronic inflammation and stunting will require more longitudinal studies, using panels of emerging biomarkers together with gut biopsy samples where feasible.

### Microbiota

Postnatal gut development is highly influenced by changes in the microbiota and diet. A recent murine study [24^▪^] showed that many genes governing intestinal development are controlled by the microbiota, while dietary shifts at weaning led to changes in metabolic and antimicrobial gene expression, indicating that intestinal development is highly influenced by the environment. The gut needs to respond readily to pathogens, while avoiding inflammation in response to the microbiota. Through a bidirectional relationship, the microbiota can drive inflammation and the mucosal inflammatory milieu shapes the microbiota. However, the microbiota remains remarkably stable due to evolution of resilience mechanisms; for example, gut commensals are resistant to the activity of intestinal antimicrobial peptides, through mechanisms that are emerging [25]. Mucosal inflammation is an important defence against enteropathogen colonization, but can also limit microbiota growth, counter-intuitively providing an advantage to pathogens that have evolved survival mechanisms [26]. Key regulators of the interactions between inflammation and the microbiota are being identified [27]. Immune cells, such as Th17, Th22 and γδ T-cells, have a critical role in intestinal homeostasis by producing interleukin (IL)-22, which maintains epithelial barrier integrity and regulates microbiota composition. IL-22-deficient mice have higher mortality than wild-type mice following *Clostridium difficile* infection due to translocation of commensals to extraintestinal organs, highlighting the importance of interactions between mucosal immune cells, intestinal barrier function and gut microbial composition in protection from pathogens [28].

The interplay between the microbiota and diarrhoeal pathogens was recently highlighted in a time-series metagenomic study of adults with cholera [29]. Recovery was associated with a pattern of changes that recapitulate the original microbiota assembly seen in healthy children, indicating that certain taxa may promote repair of the microbiota ‘organ’. In this study, one species, *Ruminococcus obeum*, reduced *Vibrio cholerae* colonization [29]. Similarly, a recent murine study [30] found that *Clostridium scindens* alone could confer colonization resistance to *C. difficile* infection, suggesting that protection from pathogens can be governed at the single species level. The protective role of the microbiota has raised concerns that perturbations of the gut community by antibiotics may impair colonization resistance. An observational study [31] of 465 children followed from birth in Vellore, India, found that those receiving antibiotics in the first 6 months of life had a 33% increased risk of diarrhoea through 3 years in adjusted analyses (incidence rate ratio 1.33, 95% CI 1.12, 1.57), although exclusive breastfeeding was protective, potentially due to beneficial bacterial species (e.g. lactobacilli) in breast milk. In the same cohort [32], children receiving antibiotics to treat diarrhoea had a subsequent diarrhoeal episode twice as soon as children not receiving antibiotics (median time ratio 0.50; 95% CI 0.38, 0.79). Although there is potential for unmeasured confounding, these studies suggest that antibiotics, particularly in young infants, may increase the risk of diarrhoea and shorten the interval between diarrhoeal episodes.

There are intriguing interrelationships between enteric and respiratory infections. For example, diarrhoea appears to increase the risk of subsequent pneumonia, possibly because of hypochlorhydria [33]. Higher gastric pH may predispose to enteric infections through loss of the protective gastric acid barrier, and increase the risk of pneumonia via reflux of heavily colonized gastric contents. An elegant murine study [34^▪^] dissected a complex mechanism through which lung infections unexpectedly cause intestinal damage. Following intranasal infection with influenza, mice developed small intestinal damage, which was not caused by viral dissemination to the gut. Instead, lung-derived CCR9^+^CD4^+^ T cells homed to the small intestine and disrupted the microbiota through interferon-gamma secretion. In response to dysbiosis, the intestinal epithelium secreted IL-15, causing Th17 polarization of mucosal CD4^+^ T cells and IL-17-mediated gut damage. Thus, infections at distant sites may disrupt intestinal homeostasis through immune-mediated effects on the microbiota; further studies in humans are needed to explore these mechanisms further.

A series of recent studies highlights the role of the microbiota in malnutrition. By constructing a microbiota ‘maturity index’ based on age-discriminatory taxa that define a healthy pattern of bacterial assembly, maturational defects in the microbiota of children with severe acute malnutrition (SAM) were identified, which were only partially and temporarily restored by nutritional rehabilitation [35^▪▪^]. Gut microbes targeted by the mucosal immune system appear particularly important, because purified immunoglobulin A (IgA)-tagged bacteria from malnourished Malawian children transmitted a weight-loss phenotype to gnotobiotic mice, and IgA responses to certain taxa, including Enterobacteriaceae, correlated with child anthropometric measures [36]. Using available metagenomic data in a secondary analysis, reduced microbiota diversity and changes in covariance network density were found to be associated with stunting severity in Malawi and Bangladesh, indicating a role of the microbiota in both linear and ponderal growth [37]. It is now apparent that the community of gut viruses (virome) emerges after birth and interacts with the bacterial microbiota [38]. Using machine-learning methods to characterize healthy assembly of the virome, children with SAM had a disrupted virome composition and, in contrast to the bacterial microbiota, community structure was not restored by therapeutic feeding [39].

### Interventions for diarrhoea, environmental enteric dysfunction and malnutrition

There is an urgent need for new approaches and scale-up of existing interventions to reduce morbidity and mortality from diarrhoea, enteropathy and malnutrition. In a systematic review [40] of nonmedical interventions, such as infrastructure investments and behaviour change communication, most showed benefits ranging from 18 to 61% reduction in diarrhoeal incidence. The Global Action Plan for the Prevention and Control of Pneumonia and Diarrhoea outlines priority, low-cost, effective interventions to end preventable pneumonia and diarrhoea deaths by 2025 [41]. A modelling exercise in South Africa showed that even 10% scale-up of 13 existing interventions for diarrhoea by 2030 would reduce under-5 diarrhoeal deaths by 48%; water, sanitation and hygiene (WASH), oral rehydration solution and exclusive breastfeeding would avert the majority of deaths [42].

Recent systematic reviews confirm the effectiveness of handwashing [43] and point-of-use water treatment [44] for diarrhoea reduction. A recent trial in Bangladesh [45] showed that safe storage of water had similar efficacy to chlorination for diarrhoea reduction; a one-time investment in a safe storage container may be more feasible than ongoing water treatment products, although its long-term effectiveness requires evaluation. Globally, 1 billion people practise open defecation and 2 billion have no access to improved sanitation. Two new trials report the impact of sanitation interventions on diarrhoea and growth. In Odisha, India, villages were randomized to latrine promotion and construction or no intervention, with subsidies for families below the poverty line [46]. Latrine coverage increased from 9 to 63% in intervention villages, but with no reduction in faecal contamination, diarrhoea, soil-transmitted helminths or malnutrition, presumably because of low coverage or usage of latrines. In Mali, villages were randomized to community-led total sanitation (CLTS) or no intervention [47^▪▪^]. CLTS employs participatory methods to sustainably eliminate open defecation and promote latrine construction using locally available materials. Access to sanitation increased by 30% in intervention villages, but there was no reduction in reported open defecation or overall diarrhoea prevalence; however, children in CLTS compared with non-CLTS villages were taller and less likely to be stunted (35 vs. 41%, respectively; prevalence ratio 0.86, 95% CI 0.74–1.0). Collectively, these studies show that increasing coverage of latrines does not necessarily change behaviour or improve health outcomes, although results from Mali suggest that WASH interventions may impact malnutrition, even without diarrhoea reduction, perhaps through an effect on EED.

Diarrhoea, pathogen carriage, microbiota composition and EED likely need to be addressed together to reduce malnutrition. Approaches focusing exclusively on feeding interventions, such as provision of lipid-based nutrient supplements, have only modest impacts on linear growth [48–50]. There is increasing appreciation that integrated approaches are needed; ongoing trials in Kenya (NCT01704105), Bangladesh (NCT01590095) and Zimbabwe [51] are evaluating the impact of combining WASH and feeding interventions to reduce stunting. Recent trials in children evaluating specific interventions for EED, including prebiotics (resistant starch type 2) [52], multiple micronutrients with or without fish oil [53] and zinc with albendazole [54], have shown little impact, although a recent trial [55] of multiple micronutrients in HIV-negative Zambian adults showed improved villous height and absorptive surface area on small intestinal biopsy after 6 weeks compared with placebo. A recent Kenyan study [56] used mesalazine to treat EED, similar to other inflammatory enteropathies. Children with SAM randomized to mesalazine, vs. placebo, had no excess adverse events and showed trends towards reduced inflammation after 28 days, providing evidence for larger efficacy trials of immunomodulation, although more potent agents targeting the small intestine in children without SAM may have greater efficacy.

Oral vaccines are the cornerstone of enteric infection prevention, but are least effective where they are most needed, possibly because of EED, enteric coinfections, malnutrition and interference from breast milk antibodies. Several strategies aimed at overcoming the oral vaccine effectiveness gap have recently been reported. In Pakistan [57], injectable poliovirus vaccine given with oral poliovirus vaccine (OPV) induced superior immune responses than OPV alone in well-nourished and malnourished infants. Withholding breastfeeding for 1 h prior to oral rotavirus vaccination paradoxically showed higher IgA seroconversion in the immediate feeding arm (37.8 vs. 28.2%; *P* = 0.07), although breast milk interference occurred in a subset of infants [58]. A trial in Karachi [59] showed no improvement in serconversion with later or additional rotavirus vaccine doses. Further studies of alternative strategies are, therefore, needed to improve oral vaccine performance in settings with the highest enteric disease burdens.

## CONCLUSION

**FIGURE 1:**
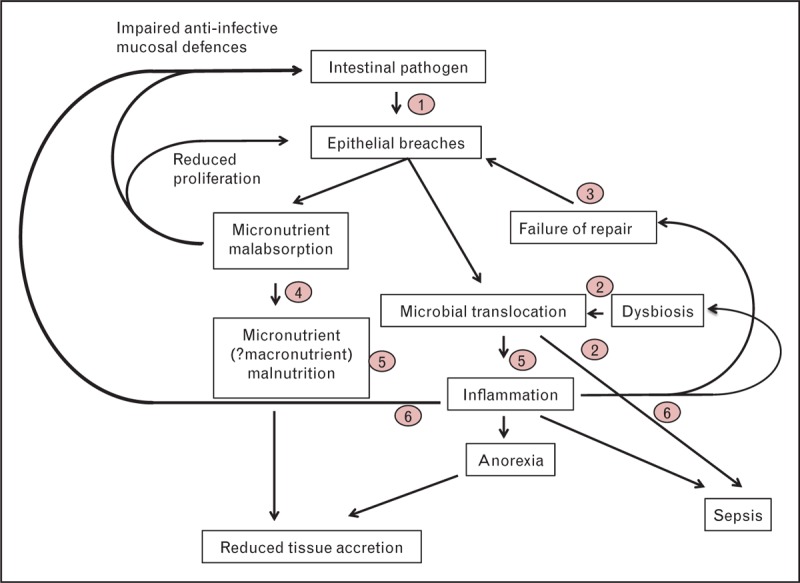
Interactions between intestinal pathogens, enteropathy and malnutrition. Contamination of the environment leads to increased exposure to intestinal pathogens that damage the mucosa and, because of failure to repair the damage, lead to microbial translocation, inflammation/sepsis and malabsorption. Several of these components reinforce mucosal damage in a positive feedback loop, exacerbating the cycle of malnutrition and infection as originally observed by Scrimshaw *et al.* in the 1960 s. Potential points for intervention include (1) water and sanitation (WASH) interventions to prevent enteropathogen exposure; (2) antimicrobial, probiotic or prebiotic agents to reduce gut colonization/dysbiosis; (3) factors (still unidentified) to enhance mucosal healing; (4) specific micronutrient supplementation to overcome specific absorptive defects, such as zinc to promote Paneth cell function; (5) anti-infective agents to reduce microbial translocation and prevent sepsis; and (6) anti-inflammatory interventions. This list is far from exhaustive.

Efforts to improve child survival and long-term developmental potential need to better understand and address the overlapping and interacting effects of diarrhoea, enteropathy and malnutrition. We believe that a pathological cycle emerges (Fig. 1), whereby intestinal pathogens cause dysbiosis and gut damage; the resulting chronic inflammation and malabsorption drive malnutrition, together with failed mucosal repair mechanisms, which render the child susceptible to further intestinal infections. Once established, this cycle may be difficult to interrupt; however, recent insights from human and animal studies suggest potential targets for intervention (Fig. 1) to reduce morbidity and mortality and improve the long-term potential of children in developing countries.

## Acknowledgements

None.

### Financial support and sponsorship

A.J.P. is funded by the Wellcome Trust (108065/Z/15/Z).

### Conflicts of interest

There are no conflicts of interest.

## REFERENCES AND RECOMMENDED READING

Papers of particular interest, published within the annual period of review, have been highlighted as:

▪ of special interest
▪▪ of outstanding interest

## Figures and Tables

**Table 1:** Comparison of the Global Enteric Multisite Study and the Malnutrition and the Consequences for Child Health and Development study

	GEMS [2^▪▪^]	Mal-ED [4^▪▪^]
Setting	Africa (Kenya, Mali, Mozambique and The Gambia) and Asia (Bangladesh, India and Pakistan)	Africa (South Africa and Tanzania), Asia (Bangladesh, India, Nepal and Pakistan) and South America (Peru and Brazil)
Ages	0–59 months	Birth cohort, recruited within 17 days of age and followed for 24 months
Clinical presentation	Children presenting to health centres with moderate-to-severe diarrhoea^a^	Diarrhoea identified during twice-weekly home visits
Design	Matched case–control study, with calculation of adjusted population attributable fractions	Comparison of diarrhoeal specimens with non-diarrhoeal surveillance specimens (collected at 1–12, 15, 18, 21 and 24 months of age), with calculation of adjusted attributable fractions
Sample size	9439 cases and 13 129 controls	2145 children
Investigations	Conventional stool culture, with PCR to further identify *Escherichia coli* Immunoassays for rotavirus, adenovirus, *Giardia lamblia, Entamoeba histolytica* and *Cryptosporidium* spp. PCR for norovirus, sapovirus and astrovirus	Conventional stool culture, with PCR to further identify *Escherichia coli* Immunoassays for *Campylobacter* spp., rotavirus, adenovirus, astrovirus, *Giardia spp., Entamoeba histolytica* and *Cryptosporidium* spp. PCR for norovirus
Highest attributable fractions	Rotavirus, *Cryptosporidium*, ST-ETEC, *Shigella*	Norovirus GII, Rotavirus, *Campylobacter* spp., Astrovirus, *Cryptosporidium* spp., *Shigella* spp. (after infancy)
Other important pathogens	*Aeromonas* and *Campylobacter jejuni* (Asia), *Vibrio cholerae* O1 (Asia and Mozambique)	Bloody diarrhoea: *Campylobacter* spp. and *Shigella* spp.

Based on findings from the Global Enteric Multisite Study (GEMS) [2^▪▪^] and The Etiology, Risk Factors, and The Interactions of Malnutrition & Enteric Infections: Consequences for Child Health and Development (Mal-ED) Project [4^▪▪^].

^a^Moderate-severe diarrhoea was defined as sunken eyes; loss of skin turgor; administration or prescription of intravenous fluids; dysentery; and admission to hospital for diarrhoea or dysentery. ST-ETEC, Enterotoxigenic *Escherichia coli* producing heat-stable toxin.
